# The Effect of Marinating on Fatty Acid Composition of Sous-Vide *Semimembranosus* Muscle from Holstein-Friesian Bulls

**DOI:** 10.3390/foods11060797

**Published:** 2022-03-10

**Authors:** Katarzyna Tkacz, Urszula Tylewicz, Renata Pietrzak-Fiećko, Monika Modzelewska-Kapituła

**Affiliations:** 1 Department of Meat Technology and Chemistry, Faculty of Food Sciences, University of Warmia and Mazury in Olsztyn, Plac Cieszyński 1, 10-719 Olsztyn, Poland; monika.modzelewska@uwm.edu.pl; 2 Department of Agricultural and Food Sciences, Alma Mater Studiorum, University of Bologna, Piazza Goidanich 60, 47521 Cesena, Italy; 3 Interdepartmental Centre for Agri-Food Industrial Research, Alma Mater Studiorum, University of Bologna, Via Quinto Bucci 336, 47521 Cesena, Italy; 4 Department of Commodities and Food Analysis, Faculty of Food Sciences, Plac Cieszyński 1, 10-719 Olsztyn, Poland; renap@uwm.edu.pl

**Keywords:** beef, fatty acids, marinating, sous-vide

## Abstract

The aim of the study was to evaluate the effect of two commercial oil marinades on marinated bovine *semimembranosus* muscles’ (*n* = 12) fatty acid composition. Fatty acids were determined in unmarinated raw and sous-vide beef and marinated muscles with two different marinades. The application of marinating changed the fatty acid composition in sous-vide beef. The sum of saturated fatty acids (SFA) and n-6/n-3 ratio decreased. However, the sum of monounsaturated fatty acids (MUFA) and polyunsaturated fatty acids (PUFA), including n-6 and n-3, increased in marinated sous-vide beef, while a proportion of conjugated linoleic acid (CLA) and arachidonic acid (AA) decreased. The concentration (mg/100 g) of the sum of SFA and CLA in sous-vide beef was unaffected by marinating; however, the treatment significantly increased the sum of MUFA, PUFA, n-6 fatty and n-3 fatty acid concentrations. Using marinades containing canola oil and spices prior to the sous-vide treatment of beef was effective in improving its fatty acid composition.

## 1. Introduction

Meat is regarded as an excellent source of protein, characterized by a good balance of essential amino acids and a high biological value, as well as a source of valuable trace minerals (iron, zinc, selenium, iodine, magnesium, potassium and sodium) and bioavailable vitamins (A, B_12_, folic acid, etc.) [1,2,3]. Considering its chemical composition, exogenic compounds and a high level of aroma and taste precursors, beef is one of the most valuable meat types in terms of nutritional value and culinary properties [4,5,6].

Both in Poland and worldwide, a part of beef, which is available on the market, originates from milk breeds, breeds of a dual purpose or from crossbreeds [6,7]. In Poland, among milk breeds, the Holstein-Friesian breed is one of the highest importance. Unfortunately, the meat obtained from such herds is often described as that of a low and unpredicted quality [5,7]. Therefore, there is a need to seek ways for improving the quality of this raw material. In our research team, we focused on exploring possibilities to obtain beef from Holstein-Friesian bulls that is as valuable as possible in terms of nutritional and culinary features [8,9,10,11,12]. It was noted that the cooking of beef muscles, such as *longissimus lumborum* (LL) and *semimembranosus* (SM), at a low temperature in vacuum packages (sous-vide) beneficially affected the meat quality in terms of sensory quality, including color, tenderness and juiciness. Moreover, sous-vide cooked LL and SM muscles had a similar quality, which is especially important taking into consideration that SM is less tender than LL if subjected to a traditional thermal treatment [10]. A similar finding was reported also by other authors investigating the influence of sous-vide on meat quality, who highlighted that a mild heating under a vacuum at low temperatures ranging from 50 °C to 85 °C for a long time (most frequently up to 48 h) [6,13,14,15,16] results in obtaining very tender and juicy products. This is possible due to the use of the temperature which is high enough to make collagen soluble (tenderness improvement) and inactivate micro-organisms [17,18]; at the same time, it is low enough to prevent an excessive denaturation of muscle proteins, which beneficially affects meat juiciness, and protects water soluble nutrients [6,15,19]. Moreover, a vacuum package enables aroma and taste compounds to concentrate in a final product [20]. The uniqueness of the thermal exchange that takes place in this method (low temperature—vacuum—reduced pressure—saturated steam), allows the cell structure to be preserved in the finished products by a reduction in the scope of protein–protein interactions and their gelation. It also allows a modification of compounds that shape the taste and aroma, and maintain a high bioavailability of amino acids [15,20]. As a result of sous-vide cooking, high sensory quality products are obtained, which are characterized by a uniform and consistent structure and a higher nutritional value (vitamins and minerals) as compared to those obtained with the use of other, including traditional, cooking methods. Additionally, sous-vide enables the increase in meat products’ shelf-life by reducing lipid oxidation and decreasing excessive cooking losses [6,14,15,20]. The advantage of sous-vide cooking over traditional cooking methods is the possibility to obtain tender meat regardless of the animal species, age and muscle type [14,20]. As a result, sous-vide cooking is nowadays widely used in restaurants and households, as well as has attained attention from the meat industry as a method for obtaining products characterized by good tenderness and juiciness [14,16]. Moreover, the sous-vide method can improve the organization of the production system in restaurants by simplifying and/or shortening the preparation time of dishes and avoiding losses related to the disposal of unsold food. This method is also used in the food industry in the development of safe, gourmet food products with improved nutritional properties [15].

To satisfy requirements of those consumers who are aware of relationships between diet, health and well-being, and looking for products which have not only an attractive appearance, taste and consistency but also a high nutritional value [2,21], studies which are aimed at searching technological treatments to obtain such products seem relevant. In our previous study, we investigated an impact of sous-vide on the composition of fatty acids in beef [9], along with other compounds important from a nutritional perspective in that they determine the nutritional value of meat and meat products [1,22] and have a great impact on human health [23,24,25]. Afterwards, in the next stage of our research, marinating as a method for improving sensory attractiveness, mainly taste and aroma acceptability of sous-vide cooked beef, was applied [12]. Four commercially available marinades were used, containing red and black pepper, garlic, onion and tomato, which positively affected sous-vide beef eating quality by improving its taste, tenderness, juiciness and appearance, as well as reducing cooking losses. The marinades which worked the best by providing beef with the most acceptable taste, the highest juiciness and overall acceptance, were Old Polish and Bordeaux [12]. In these marinades, spices such as garlic, black and red pepper having a great antioxidant potential were included. Antioxidants might inhibit lipid oxidation in meat during processing and therefore might beneficially affect the nutritional value of a final product [2,26]. This is vitally important since meat and meat products might be a good source of long-chain fatty acids n-3; the consumption of which is currently regarded as low [1]. According to our best knowledge, there is a shortage of reports focused on the effects of marinating on fatty acid compositions; therefore, to fill the gap, a study aimed at investigating the influence of Old Polish and Bordeaux marinades on fatty acids in sous-vide beef was conducted.

## 2. Materials and Methods

### 2.1. Meat Origin and Sample Preparation

In the study, *semimembranosus* (SM) muscles originated from the carcasses (*n* = 12) of young Holstein-Friesian black and white variety bulls (slaughtered at an average age of 20 months ± 2 months, body weight before slaughter about 600 kg) were used. The animal research protocol was approved by the University of Warmia and Mazury Ethics Committee (Decision No. 8/2020). Briefly, young bulls were reared in a traditional system, using milk replacer, hay and concentrate, under controlled conditions in the Agricultural Experiment Station in Bałcyny (Poland). All details about the feeding system and the slaughter procedure are described in the article by Tkacz et al. [12].

The beef carcasses were cooled for 24 h and then dissected. The muscles used in this study were cut from a left half-carcass of each animal. Muscles were transported to the Meat Technology and Chemistry Department laboratory at a refrigerated temperature in a potable refrigerator. After arrival, the muscles were vacuum packed in PA/PE 70 μm-thick pouches (Inter Arma sp. z o.o., Rudawa, Poland; total transmission rates not exceeding 10 mg/dm^2^ for model liquids, 3% acetic acid, 50% ethyl alcohol (10 days at 40 °C) and isooctane (2 days, 20 °C)) and aged until the 14th day post-mortem at 4 ± 1 °C in a climatic chamber (Memmert GmbH, Schwabach, Germany). After that, the muscles were assigned randomly to 2 groups, containing 6 muscles in each group. Each muscle was cut into three 2.5 cm-thick (average weight 200 g) steaks. One steak was regarded as a raw unmarinated control sample, another was a sous-vide cooked control sample (unmarinated sous-vide cooked) and a third was marinated and sous-vide cooked. Two commercial marinades were used: Old Polish (M1) and Bordeaux (M2) (Amco Sp.z o.o., Dybów-Kolonia, Poland). The marinades applied in this study were chosen based on previous research results, in which the most satisfactory sensory quality and a lower instrumentally evaluated tenderness were noted for Old Polish and Bordeaux marinated samples [12]. Both marinades contained black pepper, garlic, salt, canola oil and aromas. Additionally, the Old Polish marinade contained sugars and stabilizers, while the Bordeaux marinade contained red pepper and hydrolyzed plant protein. The proportions of ingredients are information proprietary for the producer. The composition, pH, viscosity and color of marinades used are shown in Table S1 (Supplementary Material). The marinating was carried out in accordance with the methodology provided by Tkacz et al. [12]. Marinades, according to the producer’s recommendation, were used in the quantity of 80 g per 1 kg of meat. The appropriate amount of marinade was weighed (8% with respect to the weight of each steak) and spread over the surface of the meat. Each sample was closed in a separate container with a lid, and then stored under refrigerated conditions (4 °C) for 24 h. After the termination of marinating, samples were weighed to determine a marinade uptake (4.34% and 4.39% for Old Polish and Bordeaux, respectively), vacuum packed separately and sous-vide cooked in Fusion Chef Julabo Diamond Z (Julabo GmbH, Seelbach, Germany) at 60 °C for 4 h, as recommended for beef [13]. After cooking, samples were cooled down in an icy water and subjected to fatty acids’ determination.

### 2.2. Fatty Acids

Fatty acids were determined according to the methodology described by Modzelewska-Kapituła [9]. Briefly, the lipid extract was prepared by cold extraction with the chloroform/methanol (2:1 *v*/*v*) according to Folch, Less and Sloane-Stanley (1957) and then, the Peisker [27] method was used for the transesterification of fatty acids with a chloroform:methanol:sulphuric acid (100:100:1) mixture. The analysis was performed using an Agilent Technologies 7890A gas chromatograph (Agilent Technologies, Inc., Santa Clara, CA, USA) with a flame-ionization detector (FID) and a 30-m long, 0.32-mm internal diameter fused silica capillary column (matrix active group: poly(ethylene glycol) phase, Supelco, Bellefonte, PA, USA) with Supelcowax 10 as a liquid phase (the film thickness was 0.25 μm). The following conditions of the separation were applied: helium as a carrier gas; 1 mL/min flow rate; detector and injector temperature 250 °C and 230 °C, respectively; column temperature 195 °C. To identify different fatty acids, their retention times were compared with Supelco™ 37 Component FAME Mix standards (Supelco, Bellefonte, PA, USA). For CLA, octadecadienoic acid, conjugated, methyl ester (Sigma-Aldrich Chemie, Steinheim am Albuch, Germany) containing a mixture of the cis- and trans-isomers of 9,11- and 10,12-octadecadienoic acid methyl esters, was used as a standard. The relative percentage (% total fatty acids) and concentration (mg/100 g) of fatty acids in beef were shown in the paper. To calculate the concentration of fatty acids in the meat (mg/100 g meat), a fat content and coefficient of 0.944 were used [28].

### 2.3. Statistical Analysis

Statistica 13.3 software (Tibco Software Inc., Tulsa, OK, USA) was used for the data analysis. Mean values were separated through Tukey honest significant difference (HSD) test, by considering a level of significance *p* < 0.05. For comparing the fatty acid composition of marinades, a variance analysis was applied.

## 3. Results

Table 1 presents the fatty acid composition of marinades used in the study. Although marinades were produced from the same components such as canola oil, salt, black pepper and garlic, they differed in terms of additives: Old Polish (M1) contained sugar and stabilizers, whereas Bordeaux (M2) contained red pepper and hydrolysed plant proteins [12]. This resulted in some variations in the fatty acid composition of marinades. The M1 marinade had a higher proportion of C16:1, C18:1, the sum of monounsaturated fatty acids (MUFA), n-6 and n-6/n-3 ratio; a lower proportion of C14:0, C16:0, C18:3 (n-3, alpha-linolenic acid, ALA), the sum of saturated fatty acids (SFA) and n-3 than M2 marinade.

Fatty acid proportions in raw unmarinated beef and beef prepared using the sous-vide method (both unmarinated control and marinated calculated from pooled data obtained from M1 and M2 marinated samples) are presented in Table 2. Both the thermal treatment and marinating affected fatty acid proportions in beef. The sous-vide treatment applied without marinating reduced the proportion of n-6 fatty acids and consequently the sum of PUFA. Generally, the application of marinating changed fatty acid composition in sous-vide beef. The C22:0 fatty acid, which was not detected either in raw or unmarinated sous-vide samples, was found in marinated sous-vide beef, due to its presence in both marinades. The marinating lowered the proportion of SFA (C14:0, C15:0, C16:0, C17:0, C18:0) in marinated sous-vide beef comparing with unmarinated raw and sous-vide beef, except for C20:0, the proportion of which increased. The proportion of MUFA such as C16:1 and C17:1 decreased in marinated samples, whereas the proportion of C18:1 and C20:1 increased in these samples. The proportion of C18:2 (n-6, linoleic acid, LA) increased in marinated sous-vide beef as compared with both raw unmarinated beef and unmarinated sous-vide beef (in which its proportion was the lowest). The increase in the proportion was noted also for ALA. On the other hand, in marinated sous-vide samples, lower proportions of C18:2 (conjugated linoleic acid, CLA) and C20:4 (n-6, arachidonic acid, AA) were found in relation to raw unmarinated beef and unmarinated sous-vide beef. As a result of these changes in fatty acid composition, the sum of SFA and the n-6/n-3 ratio decreased, whereas the sum of MUFA and polyunsaturated fatty acids (PUFA), including n-6 and n-3, increased in marinated sous-vide beef.

The influence of each marinade used on the fatty acid composition of sous-vide *semimembranosus* muscle is shown in Table 3. Significant differences between samples were noted only for three out of 16 fatty acids detected and in the sum of MUFA. In the beef samples marinated with the M2 marinade, a higher proportion of C18:1, CLA and the sum of MUFA, and a lower proportion of AA, were noted compared with M1 marinated samples. A marinade used did not differentiate the proportion of the sum of SFA, PUFA, n-3, n-6, or the n-6/n-3 ratio.

**Figure 1:**
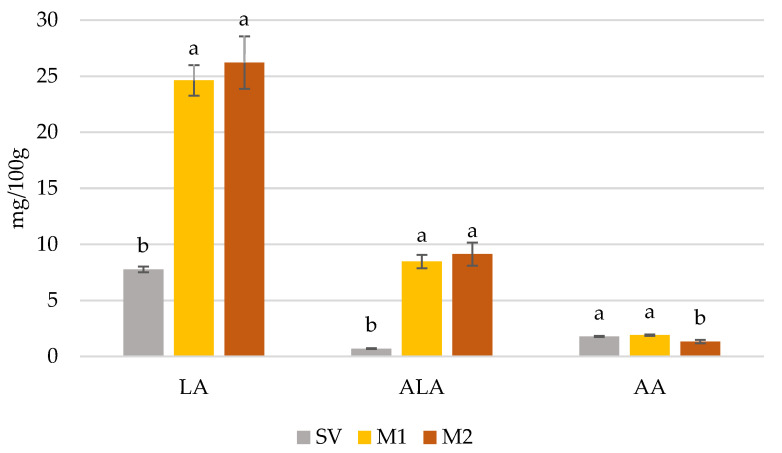
Content (mg/100 g of wet tissue) of C18:2 (n-6, LA), C18:3 (n-3, ALA) and C20:4 (n-6, AA) fatty acids in unmarinated sous-vide (SV) and marinated with M1 (Old Polish) and M2 (Bordeaux) marinades of *semimembranosus* muscle from Holstein-Friesian bulls. ^a,b^—different letters indicate significant differences at *p* < 0.05 between values for each fatty acid; vertical bars refer to standard error of the mean (SEM).

As a result of marinating, the fat content in beef increased. In unmarinated raw and sous-vide beef, fat content was 1.4% and 1.7%, respectively, whereas in M1 and M2 marinated samples it reached 2.8% and 2.4%, respectively. No differences in fat content between M1 and M2 marinated beef were noted. To show the effect of marinating using two different marinades on the nutritional value of sous-vide beef, the content of the most valuable fatty acids and the sum of SFA, MUFA, PUFA, n-3 and n-6 were calculated and expressed in mg/100 g of wet tissue. It was found that the contents of LA and ALA were similar in both marinated beef samples, and were significantly higher than in unmarinated sous-vide beef (Figure 1).

**Figure 2:**
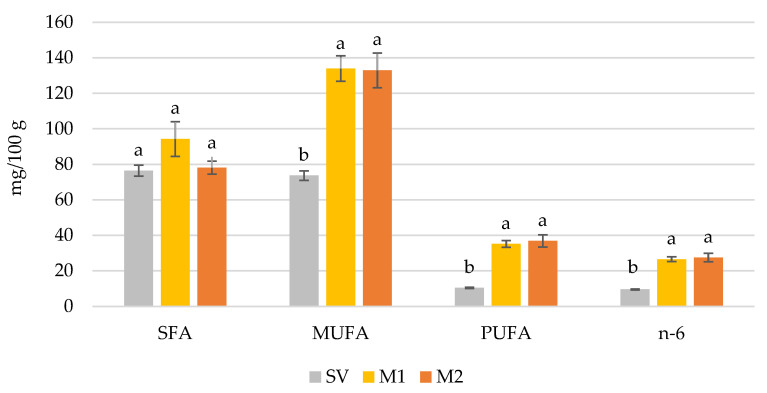
Content (mg/100g of wet tissue) of the sum of saturated (SFA), monounsaturated (MUFA), polyunsaturated (PUFA) and n-6 fatty acids in unmarinated sous-vide (SV) and marinated with M1 (Old Polish) and M2 (Bordeaux) marinades *semimembranosus* muscle from Holstein-Friesian bulls. ^a,b^—different letters indicate significant at *p* < 0.05 differences between values for each sum of fatty acid; vertical bars refer to standard error of the mean (SEM). SFA—the sum of the concentration of C14:0, C15:0, C16:0, C17:0, C18:0, C20:0, C22:0; MUFA the sum of the proportion of C14:1, C16:1, C17:1, C18:1, C20:1; PUFA the sum of the concentration of C18:2 (n-6, LA), C18:2 (CLA), C18:3 (n-3, ALA), C20:4 (n-6, AA); n-6 the sum of the concentration of C18:2 (n-6, LA) and C20:4 (n-6, AA).

Using the M2 marinade caused a significant reduction in the concentration of AA as compared with unmarinated and M1 marinated sous-vide beef (Figure 1). In the case of CLA, no significant differences between the samples were noted (0.22 mg/100 g, 0.24 mg/100 g and 0.27 mg/100 g for unmarinated sous-vide and marinated with M1 and M2 sous-vide beef, respectively). The concentration of the sum of SFA in sous-vide beef was unaffected by marinating; however, the treatment proceeded with the use of M1 and M2 marinades significantly increasing the sum of MUFA, PUFA, and n-6 and n-3 fatty acid concentrations (no differences between samples marinated with different marinades were noted) (Figure 2).

## 4. Discussion

The thermal treatment of meat obtained from different species (cattle, lamb, venison, fish) affects its fatty acid composition, which in turn reflects on its nutritional value [9,29,30,31]. In the present study, the sous-vide treatment applied without marinating decreased the proportion of the sum of PUFA, including n-6 fatty acids. PUFA are incorporated in the cell membrane structure (phospholipids), remain bound to the membrane [32] and are susceptible to oxidation [5]. The destructive impact of thermal treatment on PUFA was reported by Alfaia et al. [33], who found that thermal treatment methods such as boiling, grilling and microwave cooking decreased PUFA in beef, and by Valencak et al. [29], who also noted a decline in the PUFA content during cooking in meat of different European game animals.

In this study, a reduction in the proportion of n-6 in unmarinated sous-vide beef was noted compared with unmarinated raw meat; however, the n-3 fatty acid proportion remained unchanged in these samples. It can be explained by a higher susceptibility of n-6 fatty acids to autoxidation during cooking than n-3 [34]. In the present study, the proportion of n-3 fatty acids did not change as a result of the sous-vide treatment of unmarinated beef, which supports the findings of Campo et al. [35] about their higher oxidative stability during heating. In marinated samples, a significant increase in the proportion of MUFA and PUFA, including n-3 and n-6 fatty acids, was noted as compared with unmarinated raw and sous-vide beef. The changes observed were caused primarily by the canola oil, rich in MUFA and PUFA, present in the majority of both marinades. Additionally, spices used in the marinades, being the source of antioxidants, might have prevented PUFA from the oxidation process accelerated by a temperature of the sous-vide treatment. In the M1 and M2 marinades, garlic (being the source of such antioxidants such as allicin, diallyl sulfide, diallyl disulfide, diallyl trisulfide, allyl isothiocyanate, S-allyl cysteine); black pepper (which contains piperine, pinene, camphene, limonene, terpenes, piperidine, isoquercetin, sarmentine); and in M2 marinade, also red pepper (rich in capsaicin, tocopherol, lutein, carotene, capsanthin, quercetin, ascorbic acid) [26] were used. These antioxidants not only protect lipids and oils in food from oxidative changes, which result in the rancidity development and formation of toxic oxidation products’ inhibition, maintaining nutritional quality and extending the shelf-life of products, but also have a positive impact on human health. Garlic and black pepper show therapeutic effects, especially for cardiovascular diseases (garlic), and have been used against cancer (black pepper and garlic) [26]. The impact of marinades on CLA was studied by Manful et al. [36,37], and the protective effect of marinating with the mix of unfiltered beer, oregano, parsley, mustard, salt, pepper, garlic, olive oil, vinegar and onion towards CLA in grilled beef was noted [36]. A suppression of CLA oxidation up to 37% resulted from increased antioxidant activity in marinated grilled beef compared to the unmarinated control. Along with the increase in antioxidant activity, an increase in the phenolic content, up to 71%, was noted and a significant correlation between the phenolic contents and the antioxidant activities as well as the CLA concentration in marinated meat was found [36].

Fatty acid composition, with other important nutrients, determines the nutritional value of food products. Therefore, a lot of attention is paid to the meat and meat products’ fatty acid composition. Different groups of fatty acids adversely affect human heath: SFA increase low-density lipoprotein (LDL) cholesterol, whereas MUFA and PUFA decrease it [38], which reduces the risk of cardiovascular disease [23]. Therefore, consumers are encouraged to choose a lean meat with a high proportion of PUFA. In the present study, the application of marinating significantly increased both MUFA and PUFA proportions and concentrations in beef, thus making it more valuable from a nutritional perspective. Another indicator used to evaluate a nutritional quality of meat and meat products is the n-6/n-3 ratio. In the past, a value up to 4 was regarded as optimal [29]. However, more recently WHO/FAO [38] and EFSA [23] panels did not set specific n-3/n-6 ratio values. Nevertheless, when comparing products, lower values of the ratio indicate more beneficial fatty acid composition. In this study, a lower value of the ratio was noted in marinated sous-vide beef compared with unmarinated raw and sous-vide meat. However, there were no differences between beef marinated in different marinades, which resulted from a similar n-6/n-3 ratio in the marinades. The results obtained clearly indicate the beneficial impact of using oil marinades on fatty acid composition and n-6/n-3 ratio; therefore, this might be recommended as a way of improving the nutritional value of beef products. The recommendations for fatty acid consumption were proposed, and according to them, the total intake of SFA should not exceed 10% energy, while PUFA should account for 6–11% energy. The minimal intake levels of LA and ALA are estimated at 2.5% of energy for LA, plus 0.5% energy for ALA [39]. Treating beef before the sous-vide with marinades increased PUFA, including the LA and ALA concentrations, and made the meat more nutritious in light of the above presented recommendations.

The cis-9, trans-11 isomer of conjugated linoleic acid (CLA) (rumenic acid, RA) is one of the functional food components that may benefit health maintenance and aid in the prevention of chronic diseases due to its anti-carcinogenic and anti-atherogenic effects, which were shown in studies with animal models [40]. This precious fatty acid is present in ruminants’ milk and meat as a result of the rumen biohydrogenation of polyunsaturated fatty acids found naturally in grains and forages [40]. In the present study, the application of marinating significantly decreased the relative proportion of CLA in marinated beef as compared with its unmarinated raw and sous-vide counterparts. This resulted from the incorporation into marinated beef fatty acids such as C18:1, LA and ALA; the proportions of which in the marinades were the highest. However, this did not reflect the concentration (mg/100 g) of CLA in beef and no changes between unmarinated sous-vide and marinated with different marinades’ samples were noted.

LA and ALA are essential fatty acids since they are not synthesized in the human body. ALA is a precursor of eicosapentaenoic (EPA) and docosahexaenoic (DPA) acids, whereas LA is a precursor of arachidonic (AA) acid; these are also regarded as essential fatty acids [41]. LA and ALA are found in vegetable oils, including canola oil, whereas EPA and DHA are found in higher concentrations in marine sources (fish oils, algae, etc.). The main health benefits of PUFA came from EPA and DHA, which might exert a beneficial impact on human health by reducing inflammation and lowering a risk of chronic diseases (such as heart disease, cancer, arthritis). They also regulate blood pressure, hematic clotting, glucose tolerance and nervous system development and functions [25]. However, since the main sources of EPA and DHA (fish and other marine products) in the Western diet are consumed in lower quantities than vegetables and vegetable oil, ALA from vegetable oils needs to be converted in the body to EPA and DHA [25]. LA and ALA were abundant in both marinades applied in this study and come from canola oil. As a result, the proportions and concentrations of LA and ALA increased in sous-vide beef subjected to marinating regardless of the marinade used. Therefore, the consumption of marinated sous-vide beef might enrich a diet with this EPA and DHA precursor. LA is the precursor of AA, which is required for the functioning of cells, muscles, immune and nervous systems [42]. The AA fatty acid was not detected in either marinade, and consequently its relative proportion in marinated beef decreased. However, its proportion and concentration were higher in M1 than M2 marinated beef. AA belongs to n-6 fatty acids which, as mentioned above, are susceptible to oxidation during meat cooking. The changes between marinated samples might be explained by the protective effect of M1 marinade components on this fatty acid. While the components of the marinades were similar, they might be used in different proportions, which affected their antioxidant effectiveness.

## 5. Conclusions

In the previous study, we found a beneficial effect of marinades on beef tenderness and sensory quality. Apart from these important features, another determinant of food quality is its fatty acid composition, which was investigated in the present study. It was found that by using canola oil-based marinades containing spices (garlic, black pepper and red pepper), the fatty acid composition in sous-vide beef was improved in relation to the unmarinated control. It was demonstrated in a significant increase in the sum of MUFA, PUFA, and n-6 fatty and n-3 acids concentrations. The n-6/n-3 ratio was also reduced to a low, appreciated from a nutritional point of view, level. The marinades used differed slightly in the fatty acid composition; therefore, their impact on the concentration was similar, except for arachidonic acid, of which a higher concentration was found in M1 marinated beef than M2 samples. These observations indicate that marinating has a positive impact on the fatty acid composition of beef, and therefore might be recommended to produce ready-to-eat meat products. Nevertheless, a further study is needed to determine changes in fatty acid composition in marinated sous-vide beef intended for consumption after prolonged cold storage and reheating.

## Figures and Tables

**Table 1:** Fatty acid composition (% of total fatty acids) of M1 (Old Polish) and M2 (Bordeaux) marinades (mean values ± standard error of the mean).

Fatty Acid (% of Total)	M1	M2
C14:0	0.108 ^b^ ± 0.0002	0.149 ^a^ ± 0.0003
C16:0	5.5 ^b^ ± 0.04	6.49 ^a^ ± 0.029
C17:0	0.053 ± 0.002	0.0548 ± 0.0013
C18:0	2.28 ± 0.18	2.71 ± 0.017
C20:0	0.66 ± 0.03	0.683 ± 0.0017
C22:0	0.685 ± 0.109	0.873 ± 0.024
C16:1	0.28 ^a^ ± 0.003	0.224 ^b^ ± 0.001
C17:1	0.072 ± 0.008	0.081 ± 0.013
C18:1	63.17 ^a^ ± 0.15	61.96 ^b^ ± 0.05
C20:1	1.28 ± 0.01	1.29 ± 0.01
C22:1	0.188 ± 0.010	0.271 ± 0.029
C18:2 (n-6, LA)	18.19 ± 0.11	17.42 ± 0.01
C18:3 (n-3, ALA)	7.53 ^b^ ± 0.07	7.80 ^a^ ± 0.02
Σ SFA	9.3 ^b^ ± 0.4	10.96 ^a^ ± 0.07
Σ MUFA	65.00 ^a^ ± 0.17	63.83 ^b^ ± 0.10
Σ PUFA	25.71 ± 0.19	25.22 ± 0.03
Σ n-6	18.19 ^a^ ± 0.11	17.42 ^b^ ± 0.01
Σ n-3	7.53 ^b^ ± 0.07	7.80 ^a^ ± 0.02
n-6/n-3	2.42 ^a^ ± 0.01	2.23 ^b^ ± 0.01

^a,b^—values in rows with different lower case letters differ significantly at *p* < 0.05; Σ SFA the sum of the proportion of C14:0, C16:0, C17:0, C18:0, C20:0, C22:0; Σ MUFA the sum of the proportion of C16:1, C17:1, C18:1, C20:1, C22:1; Σ PUFA the sum of the proportion of C18:2 (n-6, LA), C18:3 (n-3, ALA); Σ n-6 the proportion of C18:2 (n-6, LA); Σ n-3 the proportion of C18:3 (n-3, ALA).

**Table 2:** The influence of sous-vide (SV) treatment and marinating on fatty acid composition (% of total fatty acids) of beef (mean values ± standard error of the mean).

Fatty Acid (% of Total)	Raw Unmarinated Beef	Unmarinated SV Beef	Marinated SV Beef
C14:0	2.56 ^a^ ± 0.11	2.66 ^a^ ± 0.12	1.70 ^b^ ± 0.11
C15:0	0.336 ^a^ ± 0.013	0.357 ^a^ ± 0.013	0.225 ^b^ ± 0.014
C16:0	27.3 ^a^ ± 0.4	27.9 ^a^ ± 0.5	19.19 ^b^ ± 0.7
C17:0	0.941 ^a^ ± 0.028	0.979 ^a^ ± 0.026	0.613 ^b^ ± 0.028
C18:0	15.11 ^a^ ± 0.24	15.5 ^a^ ± 0.4	10.7 ^b^ ± 0.4
C20:0	0.093 ^b^ ± 0.009	0.106 ^b^ ± 0.011	0.403 ^a^ ± 0.021
C22:0	ND	ND	0.77 ± 0.05
C14:1	0.504 ^a^ ± 0.028	0.50 ^a^ ± 0.04	0.31 ^b^ ± 0.04
C16:1	3.82 ^a^ ± 0.10	3.76 ^a^ ± 0.18	2.36 ^b^ ±0.15
C17:1	0.812 ^a^ ± 0.021	0.807 ^a^ ± 0.025	0.468 ^b^ ± 0.023
C18:1	39.6 ^b^ ± 0.5	40.5 ^b^ ± 0.5	48.4 ^a^ ± 0.7
C20:1	0.32 ^b^ ± 0.04	0.36 ^b^ ± 0.04	0.758 ^a^ ± 0.021
C18:2 (n-6, LA)	6.2 ^b^ ± 0.4	4.87 ^c^ ± 0.14	9.99 ^a^ ± 0.28
C18:2 (CLA)	0.098 ^a^ ± 0.006	0.093 ^a^ ± 0.007	0.069 ^b^ ± 0.005
C18:3 (n-3, ALA)	0.55 ^a^ ± 0.03	0.434 ^a^ ± 0.019	3.45 ^b^ ± 0.18
C20:4 (n-6, AA)	1.77 ^a^ ± 0.16	1.13 ^b^ ± 0.05	0.64 ^c^ ± 0.05
Σ SFA	46.3 ^a^ ± 0.4	47.6 ^a^ ± 0.6	33.6 ^b^ ± 1.1
Σ MUFA	45.1 ^b^ ± 0.5	45.9 ^b^ ± 0.5	52.3 ^a^ ± 0.6
Σ PUFA	8.6 ^b^ ± 0.6	6.52 ^c^ ± 0.18	14.2 ^a^ ± 0.6
Σ n-6	8.0 ^b^ ± 0.6	6.0 ^c^ ± 0.17	10.6 ^a^ ± 0.4
Σ n-3	0.55 ^b^ ± 0.03	0.434 ^b^ ± 0.019	3.45 ^a^ ± 0.18
n-6/n-3	14.7 ^a^ ± 0.8	14.0 ^a^ ± 0.6	3.1 ^b^ ± 0.1

^a,b,c^—values in rows with different lower case letters differ significantly at *p* < 0.05; ND—not detected; Σ SFA the sum of the proportion of C14:0, C15:0, C16:0, C17:0, C18:0, C20:0, C22:0; Σ MUFA the sum of the proportion of C14:1, C16:1, C17:1, C18:1, C20:1; Σ PUFA the sum of the proportion of C18:2 (n-6, LA), C18:2 (CLA), C18:3 (n-3, ALA), C20:4 (n-6, AA); Σ n-6 the sum of the proportion of C18:2 (n-6, LA) and C20:4 (n-6, AA); Σ n-3 the proportion of C18:3 (n-3, ALA).

**Table 3:** Comparison of fatty acid composition (% of total fatty acids) of sous-vide (SV) beef marinated using M1 (Old Polish) and M2 (Bordeaux) marinades (mean values ± standard error of the mean).

Fatty Acid (% of Total)	M1	M2
C14:0	1.85 ± 0.17	1.54 ± 0.13
C15:0	0.220 ± 0.019	0.231 ± 0.021
C16:0	20.5 ± 1.1	17.9 ± 0.6
C17:0	0.62 ± 0.04	0.61 ± 0.04
C18:0	10.9 ± 0.4	10.4 ± 0.8
C20:0	0.40 ± 0.03	0.409 ± 0.026
C22:0	0.85 ± 0.09	0.70 ± 0.03
C14:1	0.32 ± 0.04	0.30 ± 0.06
C16:1	2.46 ± 0.22	2.27 ± 0.21
C17:1	0.49 ± 0.04	0.451 ± 0.02
C18:1	47.1 ^b^ ± 0.9	49.7 ^a^ ± 0.7
C20:1	0.749 ± 0.028	0.7 ± 0.03
C18:2 (n-6, LA)	9.5 ± 0.6	10.51 ± 0.4
C18:2 (CLA)	0.057 ^b^ ± 0.004	0.081 ^a^ ± 0.008
C18:3 (n-3, ALA)	3.26 ± 0.26	3.64 ± 0.24
C20:4 (n-6, AA)	0.75 ^a^ ± 0.06	0.54 ^b^ ± 0.05
Σ SFA	35.4 ± 1.5	31.7 ± 8 1.1
Σ MUFA	51.1 ^b^ ± 0.7	53.5 ^a^ ± 0.5
Σ PUFA	13.5 ± 0.9	14.8 ± 0.6
Σ n-6	10.2 ± 0.7	11.0 ± 0.4
Σ n-3	3.26 ± 0.26	3.64 ± 0.24
n-6/n-3	3.2 ± 0.1	3.1 ± 0.1

^a,b^—values in rows with different lower case letters differ significantly at *p* < 0.05; Σ SFA the sum of the proportion of C14:0, C15:0, C16:0, C17:0, C18:0, C20:0, C22:0; Σ MUFA the sum of the proportion of C14:1, C16:1, C17:1, C18:1, C20:1; Σ PUFA the sum of the proportion of C18:2 (n-6, LA), C18:2 (CLA), C18:3 (n-3, ALA), C20:4 (n-6, AA); Σ n-6 the sum of the proportion of C18:2 (n-6, LA) and C20:4 (n-6, AA); Σ n-3 the proportion of C18:3 (n-3, ALA).

## Data Availability

Reasonable requests for datasets generated from the current experiment are available from the corresponding authors.

## Supplementary Materials

The following supporting information can be downloaded at: https://www.mdpi.com/article/10.3390/foods11060797/s1, Table S1: The composition, color, pH and viscosity of commercial marinates used in the study, Figure S1: Marinade Old Polish, Figure S2: Marinade Bordeaux.

## References

[1] Bohrer B.M. (2017). Review: Nutrient density and nutritional value of meat products and non-meat foods high in protein. Trends Food Sci. Technol..

[2] Das A.K., Nanda P.K., Madane P., Biswas S., Das A., Zhang W., Lorenzo J.M. (2020). A comprehensive review on antioxidant dietary fibre enriched meat-based functional foods. Trends Food Sci. Technol..

[3] Ren Q.-S., Fang K., Yang X.-T., Han J.-W. (2022). Ensuring the quality of meat in cold chain logistics: A comprehensive review. Trends Food Sci. Technol..

[4] Kang N., Panzone L., Kuznesof S. (2022). The role of cooking in consumers’ quality formation: An exploratory study of beef steaks. Meat Sci..

[5] Pogorzelski G., Pogorzelska-Nowicka E., Pogorzelski P., Półtorak A., Hocquette J.-F., Wierzbicka A. (2022). Towards an integration of pre- and post-slaughter factors affecting the eating quality of beef. Livest. Sci..

[6] Supaphon P., Kerdpiboon S., Vénien A., Loison O., Sicard J., Rouel J., Astruc T. (2021). Structural changes in local Thai beef during sous-vide cooking. Meat Sci..

[7] Nogalski Z., Sobczuk-Szul M., Pogorzelska-Przybyłek P., Wielgosz-Groth Z., Purwin C., Modzelewska-Kapituła M. (2016). Comparison of slaughter value for once-calved heifers and heifers of Polish Holstein-Friesian × Limousine crossbreds. Meat Sci..

[8] Modzelewska-Kapituła M., Tkacz K., Nogalski Z., Karpińska-Tymoszczyk M., Draszanowska A., Pietrzak-Fiećko R., Purwin C., Lipiński K. (2018). Addition of herbal extracts to the Holstein-Friesian bulls’ diet changes the quality of beef. Meat Sci..

[9] Modzelewska-Kapituła M., Pietrzak-Fiećko R., Tkacz K., Draszanowska A., Więk A. (2019). Influence of sous vide and steam cooking on mineral contents, fatty acid composition and tenderness of semimembranosus muscle from Holstein-Friesian bulls. Meat Sci..

[10] Modzelewska-Kapituła M., Tkacz K., Nogalski Z. (2021). The influence of muscle, ageing and thermal treatment method on the quality of cooked beef. J. Food Sci. Technol..

[11] Tkacz K., Modzelewska-Kapituła M., Więk A., Nogalski Z. (2020). The Applicability of Total Color Difference ΔE for Determining the Blooming Time in Longissimus Lumborum and Semimembranosus Muscles from Holstein-Friesian Bulls at Different Ageing Times. Appl. Sci..

[12] Tkacz K., Modzelewska-Kapituła M., Petracci M., Zduńczyk W. (2021). Improving the quality of sous-vide beef from Holstein-Friesian bulls by different marinades. Meat Sci..

[13] Baldwin D.E. (2012). Sous vide cooking: A review. Int. J. Gastron. Food Sci..

[14] Naqvi Z.B., Thomson P.C., Ha M., Campbell M.A., McGill D.M., Friend M.A., Warner R.D. (2021). Effect of sous vide cooking and ageing on tenderness and water-holding capacity of low-value beef muscles from young and older animals. Meat Sci..

[15] Kathuria D., Dhiman A.K., Attri S. (2022). Sous vide, a culinary technique for improving quality of food products: A review. Trends Food Sci. Technol..

[16] Chian F.M., Kaur L., Astruc T., Venien A., Stubler A.S., Aganovic K., Loison O., Hodgkinson S., Boland M. (2021). Shockwave processing of beef brisket in conjunction with sous-vide cooking: Effects on protein structural characteristics and muscle microstructure. Food Chem..

[17] Roldan M., Antequera T., Martín A., Mayoral A.I., Ruiz J. (2013). Effect of different temperature–time combinations on physicochemical, microbiological, textural and structural features of sous-vide cooked lamb loins. Meat Sci..

[18] Singh C.B., Kumari N., Senapati S.R., Lekshmi M., Nagalakshmi K., Balange A.K., Chouksey M.K., Venkateshwarlu G., Xavier K.A.M. (2016). Sous vide processed ready-to-cook seerfish steaks: Process optimization by response surface methodology and its quality evaluation. LWT.

[19] Botinestean C., Keenan D.F., Kerry J.P., Hamill R.M. (2016). The effect of thermal treatments including sous-vide, blast freezing and their combinations on beef tenderness of *M. semitendinosus* steaks targeted at elderly consumers. LWT.

[20] Ferigolo L.P., Elias S.O., Carmo da Silva D., Lopes S.M., Geimba M.P., Tondo E.C. (2021). Escherichia coli inactivation on tenderloin beef medallions processed by sous vide treatment. Int. J. Gastron. Food Sci..

[21] Moran L., Wilson S.S., McElhinney C.K., Monahan F.J., McGee M., O’Sullivan M.G., O’Riordan E.G., Kerry J.P., Moloney A.P. (2019). Suckler Bulls Slaughtered at 15 Months of Age: Effect of Different Production Systems on the Fatty Acid Profile and Selected Quality Characteristics of Longissimus Thoracis. Foods.

[22] Wood J.D., Enser M., Purslow P.P. (2017). Chapter 20—Manipulating the Fatty Acid Composition of Meat to Improve Nutritional Value and Meat Quality. New Aspects of Meat Quality.

[23] EFSA Panel on Dietetic Products, Nutrition, and Allergies (NDA) (2010). Scientific opinion on dietary reference values for fats, including saturated fatty acids, polyunsaturated fatty acids, monounsaturated fatty acids, trans fatty acids, and cholesterol. EFSA J..

[24] Silva L.F., Barbosa A.M., da Silva Júnior J.M., Oliveira V.d.S., Gouveia A.A.L., Silva T.M., Lima A.G.V.D.O., Nascimento T.V.C., Bezerra L.R., Oliveira R.L. (2022). Growth, physicochemical properties, fatty acid composition and sensorial attributes from longissumus lumborum of young bulls fed diets with containing licuri cake: Meat quality of bulls fed licuri cake. Livest. Sci..

[25] Gammone M.A., Riccioni G., Parrinello G., D’orazio N. (2019). Omega-3 polyunsaturated fatty acids: Benefits and endpoints in sport. Nutrients.

[26] Yashin A., Yashin Y., Xia X., Nemzer B. (2017). Antioxidant Activity of Spices and Their Impact on Human Health: A Review. Antioxidants.

[27] Peisker K. (1964). A rapid semi-micro method for preparation of methyl esters from triglycerides using chloroform, methanol, sulphuric acid. J. Am. Oil Chem. Soc..

[28] Kunachowicz H., Nadolna I., Przygoda B., Iwanow K. (2005). Tables of Nutritional Value of Food Products and Dishes.

[29] Valencak T.G., Gamsjäger L., Ohrnberger S., Culbert N.J., Ruf T. (2015). Healthy n-6/n-3 fatty acid composition from five European game meat species remains after cooking. BMC Res. Notes.

[30] Pietrzak-Fiećko R., Modzelewska-Kapituła M., Zakęś Z., Szczepkowski M. (2017). The Effect of Thermal Treatment Method on Fatty Acid Composition in Northern Pike (*Esox lucius*) Fillets. J. Aquat. Food Prod. Technol..

[31] Suleman R., Wang Z., Aadil R.M., Hui T., Hopkins D.L., Zhang D. (2020). Effect of cooking on the nutritive quality, sensory properties and safety of lamb meat: Current challenges and future prospects. Meat Sci..

[32] Gerber N., Scheeder M.R.L., Wenk C. (2009). The influence of cooking and fat trimming on the actual nutrient intake from meat. Meat Sci..

[33] Alfaia C.M.M., Alves S.P., Lopes A.F., Fernandes M.J.E., Costa A.S.H., Fontes C.M.G.A., Castro M.L.F., Bessa R.J.B., Prates J.A.M. (2010). Effect of cooking methods on fatty acids, conjugated isomers of linoleic acid and nutritional quality of beef intramuscular fat. Meat Sci..

[34] Kouba M., Benatmane F., Blochet J.E., Mourot J. (2008). Effect of a linseed diet on lipid oxidation, fatty acid composition of muscle, perirenal fat, and raw and cooked rabbit meat. Meat Sci..

[35] Campo M.M., Muela E., Olleta J.L., Moreno L.A., Santaliestra-Pasías A.M., Mesana M.I., Sañudo C. (2013). Influence of cooking method on the nutrient composition of Spanish light lamb. J. Food Compost Anal..

[36] Manful C.F., Vidal N.P., Pham T.H., Nadeem M., Wheeler E., Hamilton M.C., Doody K.M., Thomas R.H. (2020). Unfiltered beer based marinades reduced exposure to carcinogens and suppressed conjugated fatty acid oxidation in grilled meats. Food Control.

[37] Manful C.F., Pham T.H., Nadeem M., Wheeler E., Warren K.J.T., Vidal N.P., Thomas R.H. (2021). Assessing unfiltered beer-based marinades effects on ether and ester linked phosphatidylcholines and phosphatidylethanolamines in grilled beef and moose meat. Meat Sci..

[38] Rocha D.M., Caldas A.P., Oliveira L.L., Bressan J., Hermsdorff H.H. (2016). Saturated fatty acids trigger TLR4-mediated inflammatory response. Atherosclerosis.

[39] WHO, FAO (2008). Interim Summary of Conclusions and Dietary Recommendations on Total Fat & Fatty Acids from the Joint FAO/WHO Expert Consultation on Fats and Fatty Acids in Human Nutrition, 10–14 November 2008.

[40] Lock A.L., O’Donnell-Megaro A.M., Bauman D.E., McSweeney P.L.H., McNamara J.P. (2022). Conjugated Linoleic Acid. Encyclopedia of Dairy Sciences.

[41] Geranpour M., Assadpour E., Jafari S.M. (2020). Recent advances in the spray drying encapsulation of essential fatty acids and functional oils. Trends Food Sci. Technol..

[42] Tallima H., El Ridi R. (2018). Arachidonic acid: Physiological roles and potential health benefits—A review. J. Adv. Res..

