# Supplementary material for: The Effect of Marinating on Fatty Acid Composition of Sous-Vide Semimembranosus Muscle from Holstein-Friesian Bulls
Source: Foods. 2022 Mar 10;11(6):797. doi: 10.3390/foods11060797 (PMC8949574; doi:10.3390/foods11060797)
Supplement: Supplementary file 1 [file foods-11-00797-s001.zip › foods-1614868sup.pdf]

Table S1. The composition, colour, pH and viscosity of commercial marinates used in the study

	**Old Polish M1**	**Bordeaux M2**
	Canola oil salt aromas	
		Red pepper
	Pepper	Pepper
	Garlic	Garlic
	Sugars	Hydrolysed plant protein
	Stabilizers	
Colour		
L*	49.35 [a] (0.11)	8.54 [b] (0.06)
a*	-5.63 [b] (0.12)	16.59 [a] (0.10)
b*	43.39 [a] (0.18)	12.84 [b] (0.11)
C*	49.67 [a] (0,10)	18.66 [b] (0.09)
h˜	-83.49 [b] (0.16)	37.73 [a] (0.28)
pH		
	5.757 [a] (0.012)	5.187 [b] (0.015)
Viscosity [Pa s]		
	2.55 [b] (0.03)	3.15 [a] (0.05)
Marinades uptake [%]		
	4.34 (0.26)	4.39 (0.15)
Cooking loss [%]		
	19.7 (0.7)	18.9 (1.1)

[a-b] – mean values in rows with different superscripts differ significantly at $P < 0.05$
