# Supplementary figures and images for: The Effect of Marinating on Fatty Acid Composition of Sous-Vide Semimembranosus Muscle from Holstein-Friesian Bulls
Source: Foods. 2022 Mar 10;11(6):797. doi: 10.3390/foods11060797 (PMC8949574; doi:10.3390/foods11060797)

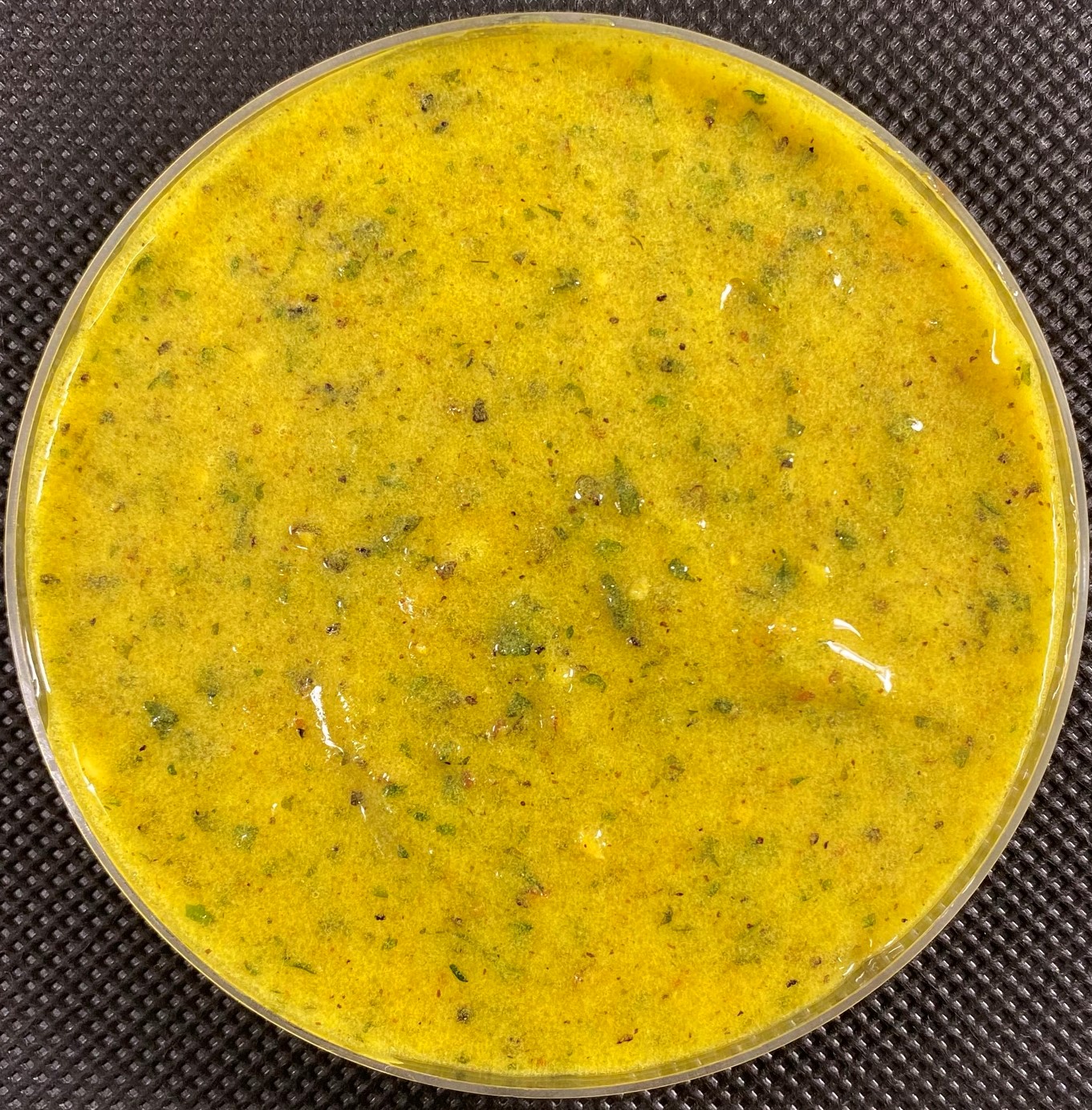

Supplement: Supplementary file 1 [file foods-11-00797-s001.zip › Picture 1. Marinade Old Polish.jpeg]

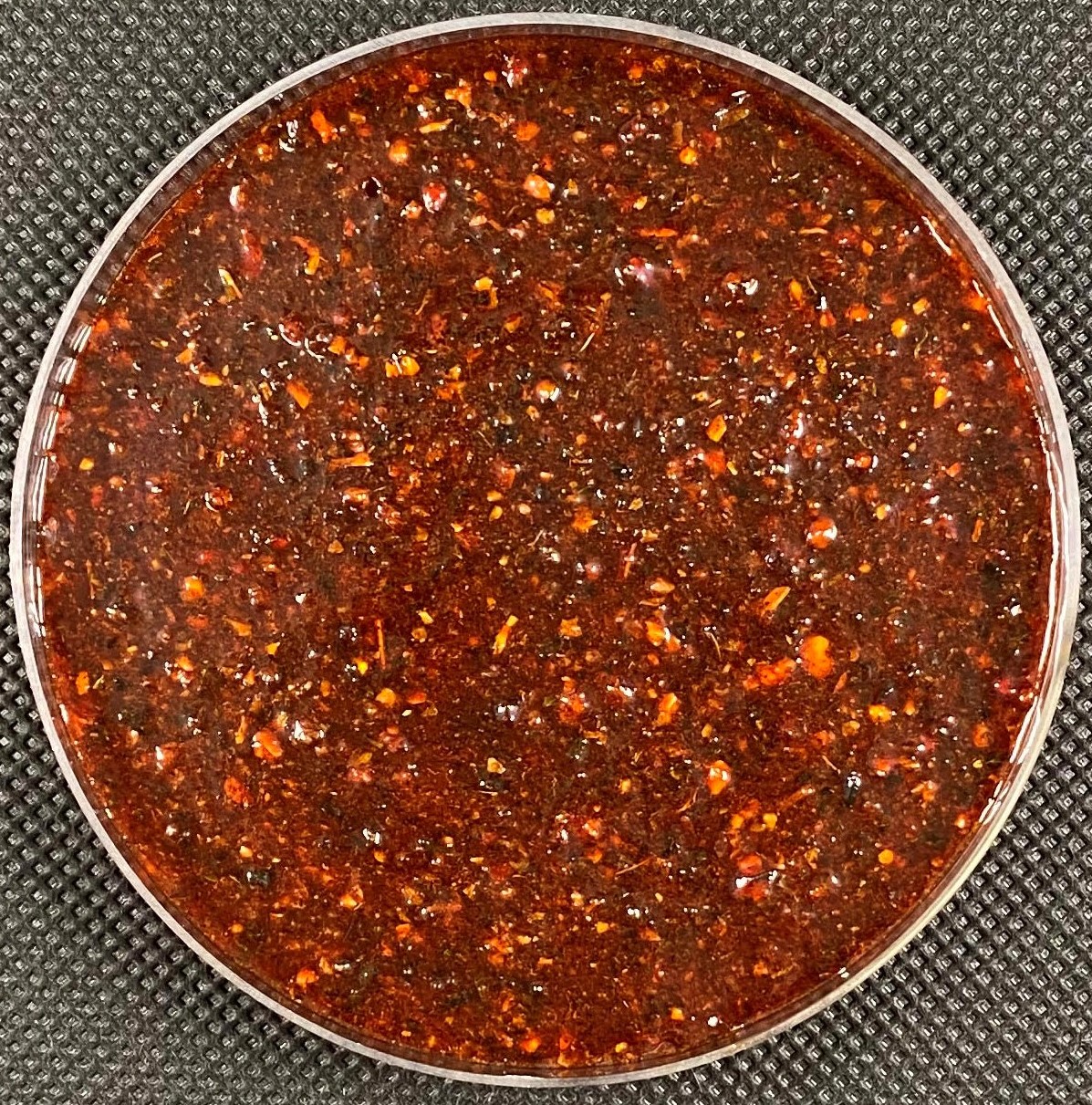

Supplement: Supplementary file 1 [file foods-11-00797-s001.zip › Picture 2. Marinade Bordeaux.jpg]
